# The Genus *Schizoprymnus* Förster, 1863 (Hymenoptera, Braconidae, Brachistinae) from China, with Descriptions of Seven New Species †

**DOI:** 10.3390/insects14010036

**Published:** 2022-12-30

**Authors:** Chengjin Yan, Qiong Wu, Cornelis van Achterberg, Xuexin Chen

**Affiliations:** 1 State Key Laboratory of Rice Biology, Zhejiang University, Hangzhou 310058, China; 2 Zhejiang Provincial Key Lab of Biology of Crop Pathogens and Insects, Institute of Insect Sciences, Zhejiang University, Hangzhou 310058, China; 3 Institute of Agriculture and Biotechnology, Wenzhou Vocational College of Science and Technology, Wenzhou Academy of Agricultural Sciences, Wenzhou 325006, China

**Keywords:** parasitoid wasps, Braconidae, Brachistinae, *Schizoprymnus*, new record, new species, China

## Abstract

**Simple Summary:**

*Schizoprymnus* is a genus of braconid wasps distributed throughout the whole world except the Neotropical region. There are 126 extant species of *Schizoprymnus*, 22 of which are known from China (without new species described in this article). This paper describes and illustrates seven new species found in China.

**Abstract:**

The species of the genus *Schizoprymnus* Förster, 1863 (Hymenoptera, Braconidae, Brachistinae) from China are revised. Seven new species, namely *S. carinatus* Yan and Chen, sp. nov., *S. glabratus* Yan and Chen, sp. nov., *S. liui* Yan and Chen, sp. nov., *S. parvidentatus* Yan and van Achterberg, sp. nov., *S. punctiscutellaris* Yan and Chen, sp. nov., *S. septentrionalis* Yan and Chen, sp. nov., and *S. subspinosus* Yan and Chen, sp. nov. are described and illustrated. In addition, *S. telengai* Tobias, 1976 is reported for the first time from China. An updated key to the Chinese species of the genus *Schizoprymnus* is included.

## 1. Introduction

The genus *Schizoprymnus* Förster, 1863 belongs to the parasitoid wasp subfamily Brachistinae Förster, 1863 (Hymenoptera, Braconidae, Brachistinae), members of which are characterized by anterior three metasomal tergites immovably fused to form a carapace and two transverse sutures of a carapace usually absent or visible only along the lateral part of the carapace [1,2,3,4,5,6]. Larvae of the beetles of the families Apionidae, Brentidae, Chrysomelidae, Curculionidae, and Mordellidae are the most frequently recorded hosts of *Schizoprymnus* species [7].

It comprises 126 extant species [7,8] distributed through the Palaearctic, Afrotropical, Oriental, Nearctic, and Australasian regions [3,4,5,6,7,8,9,10,11,12,13,14,15,16,17,18,19,20,21]. So far, 22 species are known in China [7,11,22]. In the present paper, seven new species (*S. carinatus* Yan and Chen, sp. nov., *S. glabratus* Yan and Chen, sp. nov., *S. liui* Yan and Chen, sp. nov., *S*. *parvidentatus* Yan and van Achterberg, sp. nov., *S. punctiscutellaris* Yan and Chen, sp. nov., *S*. *septentrionalis* Yan and Chen, sp. nov. and *S. subspinosus* Yan and Chen, sp. nov.) are described and illustrated in detail, and an updated key to the Chinese species of *Schizoprymnus* is supplied (updated key from Chou and Hsu, 1996). As far as is known, *Schizoprymnus* are ovo-larval koinobiont endoparasitoids of coleopterous larvae [7,10,18,23,24,25,26].

## 2. Materials and Methods

This work is mainly based on specimens of *Schizoprymnus* collected by sweeping net and mostly Malaise traps (MT) set up in China. The key to the Chinese species of the genus *Schizoprymnus* is updated manually from Chou and Hsu, 1996. The terminology and measurements used follow van Achterberg (1988, 1993). Additional sources for the description of sculpture and setation are from Belokobylskij (1998). The following abbreviations are used: POL—postocellar line; OOL—ocular-ocellar line; OD—maximum diameter of lateral ocellus.

Descriptions and measurements were made under a stereomicroscope (Zeiss Stemi SV 6, ZEISS, Oberkochen, Germany). All figures were made by a camera (QImaging, Micropublisher, 3.3 RTV, QImaging, Surrey, BC, Canada) attached to a stereomicroscope (Leica MZ APO, Leica, Wetzlar, Germany) and Auto-Montage Pro version 5.0 software.

Type specimens and other materials are deposited in the Parasitic Hymenoptera Collection of the Zhejiang University, Hangzhou, China (ZJUH).

## 3. Results

### 3.1. Generic Treatment

Genus: *Schizoprymnus* Förster, 1863.

*Schizoprymnus* Förster, 1863, 19: 242; Gibson, 1972, 8: 85; Papp, 1984, 30(1–2): 138; Belokobylskij, 1998: 441, 472–489; Belokobylskij et Maetô, 2007: 171–178; Shamim, 2012: 231–233.

*Triaspis* (*Schizoprymnus*): Fahringer, 1934, 3(5–8): 368; Martin, 1956, 965: 98. Chou et Hsu, 1996: 442.

Type species: *Sigalphus obscurus* Nees, 1816.

Diagnosis. Anterior three metasomal tergites immovably fused to form a carapace; both transverse sutures of the carapace are usually absent, but in some species of *Schizoprymnus* the carapace has the first suture almost entirely impressed and the second one laterally developed. Prepectal carina complete; tarsal claws simple or with a small acute lobe.

Host. Larvae of Coleoptera (Apionidae, Brentidae, Chrysomelidae, Curculionidae, and Mordellidae) [7,10,14,18,23,24,25,26,27,28,29,30,31,32].

Distribution. China (Jilin, Liaoning, Inner Mongolia, Ningxia, Hebei, Hunan, Fujian, Zhejiang, Sichuan, Taiwan, and Guizhou); Palaearctic, Afrotropical, Oriental, Nearctic, and Australasian regions [7].

#### Key to the Chinese Species of the Genus Schizoprymnus Förster (Updated Key from Chou and Hsu, 1996)

**Figure 1:**
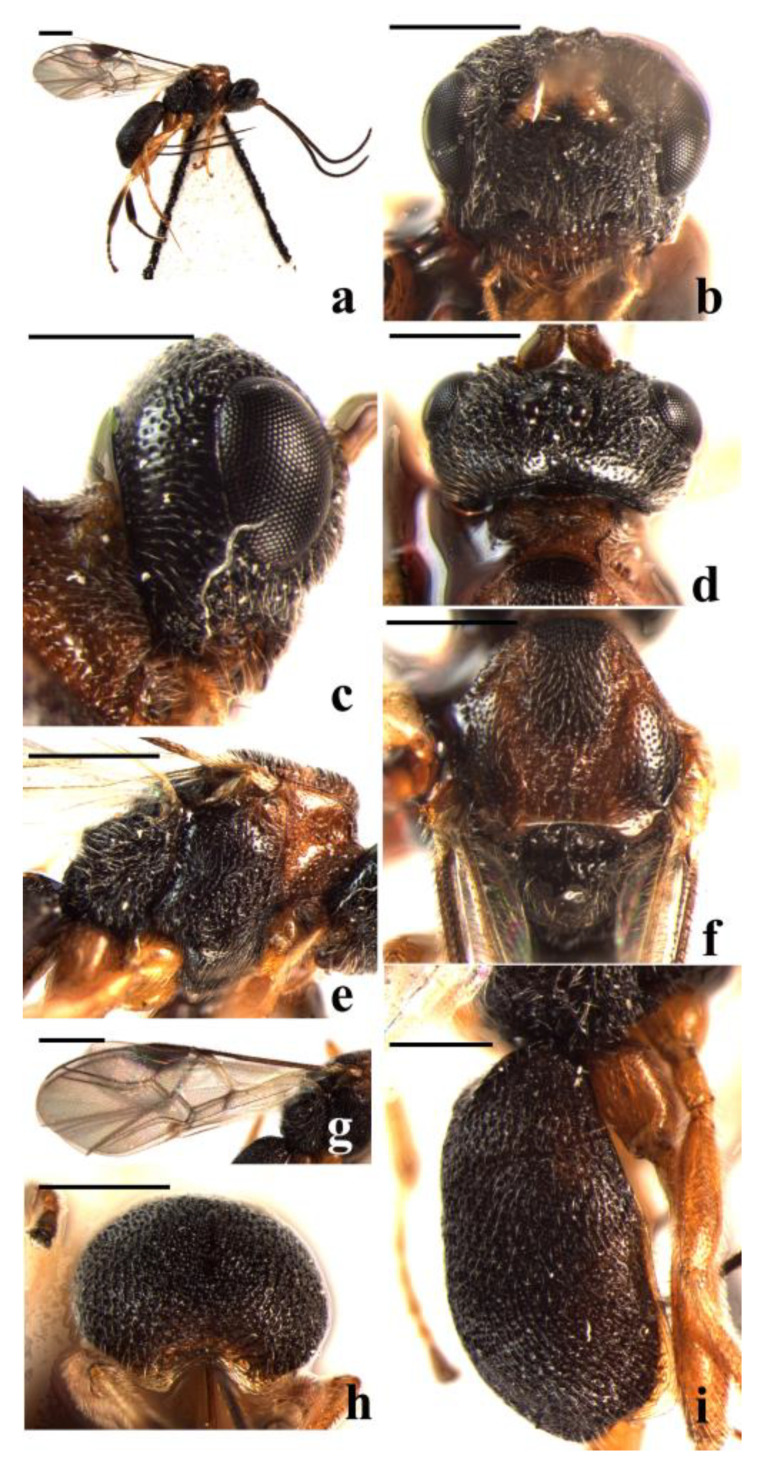
*Schizoprymnus carinatus*, sp. nov., holotype, ♀. (**a**) Habitus, lateral aspect; (**b**) head, frontal aspect; (**c**) head, lateral aspect; (**d**) head, dorsal aspect; I mesosoma, lateral aspect; (**f**) mesoscutum, dorsal aspect; (**g**) fore wing; (**h**) posterior margin of carapace; (**i**) carapace, lateral aspect. Scale bar: 1 mm.

**Figure 2:**
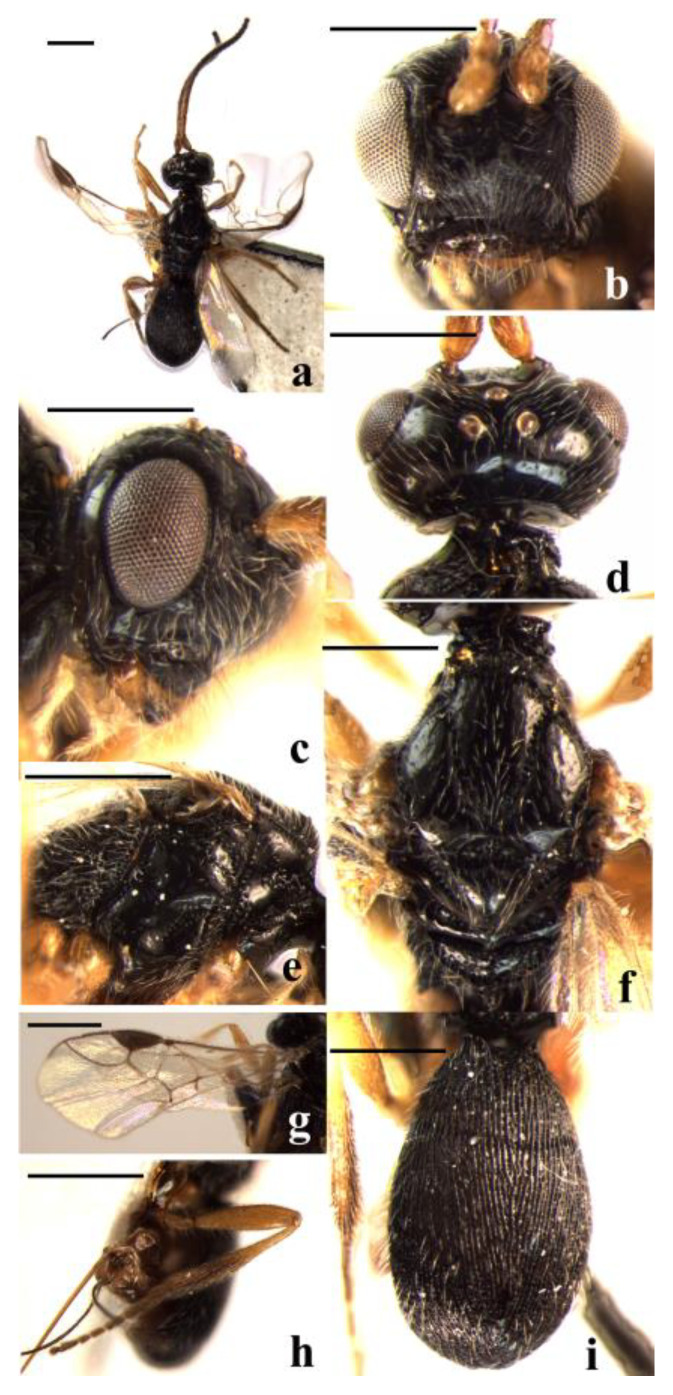
*Schizoprymnus glabratus* sp. nov., holotype, ♀. (**a**) Habitus, dorsal aspect; (**b**) head, frontal aspect; (**c**) head, lateral aspect; (**d**) head, dorsal aspect; (**e**) mesosoma, lateral aspect; (**f**) mesoscutum, dorsal aspect; (**g**) fore wing; (**h**) hind leg, lateral aspect; (**i**) carapace, dorsal aspect. Scale bar: 1 mm.

**Figure 3:**
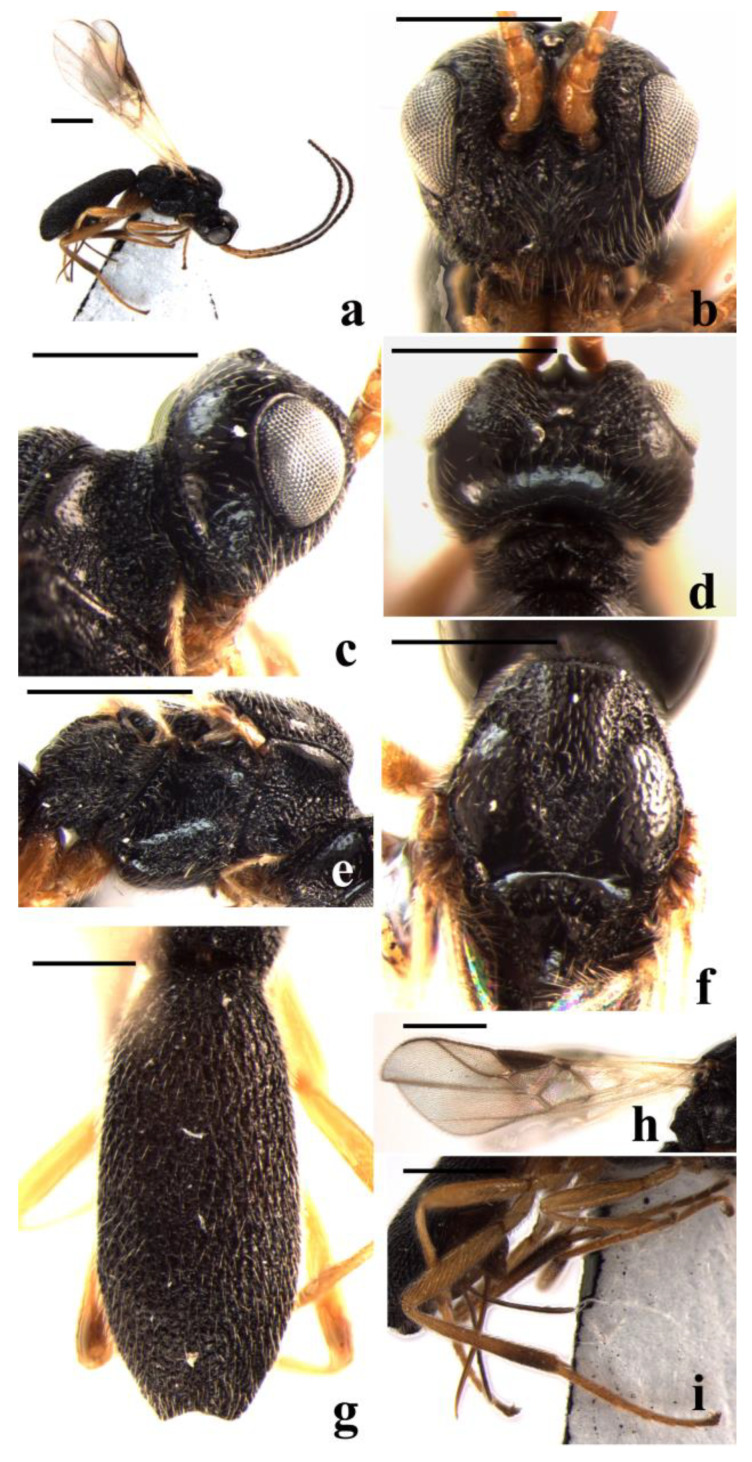
*Schizoprymnus liui* sp. nov., holotype, ♀. (**a**) Habitus, lateral aspect; (**b**) head, frontal aspect; (**c**) head, lateral aspect; (**d**) head, dorsal aspect; (**e**) mesosoma, lateral aspect; (**f**) mesoscutum, dorsal aspect; (**g**) carapace, dorsal aspect; (**h**) fore wing; (**i**) hind leg, lateral aspect. Scale bar: 1 mm.

**Figure 4:**
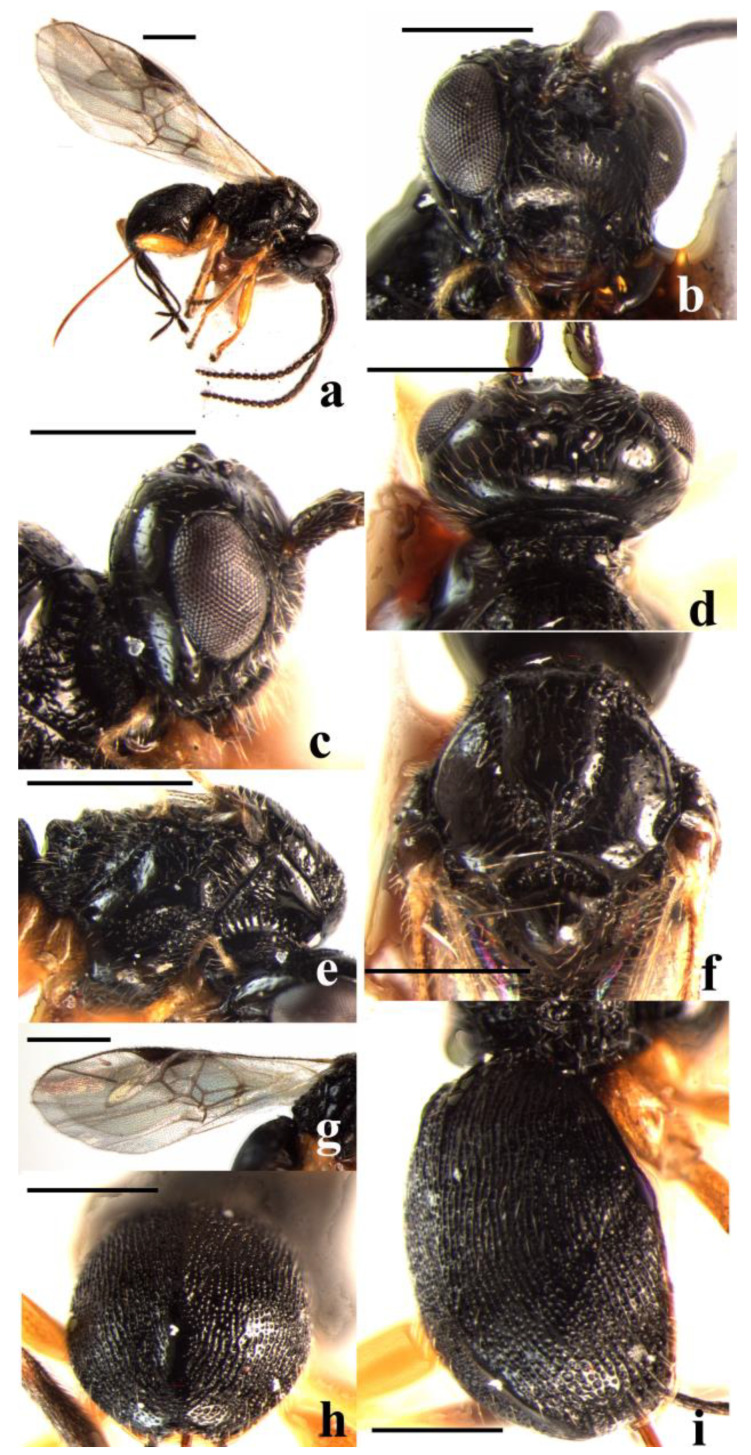
*Schizoprymnus parvidentatus* sp. nov., holotype, ♀. (**a**) Habitus, lateral aspect; (**b**) head, latero-anterior aspect; (**c**) head, lateral aspect; (**d**) head, dorsal aspect; (**e**) mesosoma, lateral aspect; (**f**) mesoscutum, dorsal aspect; (**g**) fore wing; (**h**) third metasomal tergite apically, dorsal aspect; (**i**) carapace, latero-dorsal aspect. Scale bar: 1 mm.

**Figure 5:**
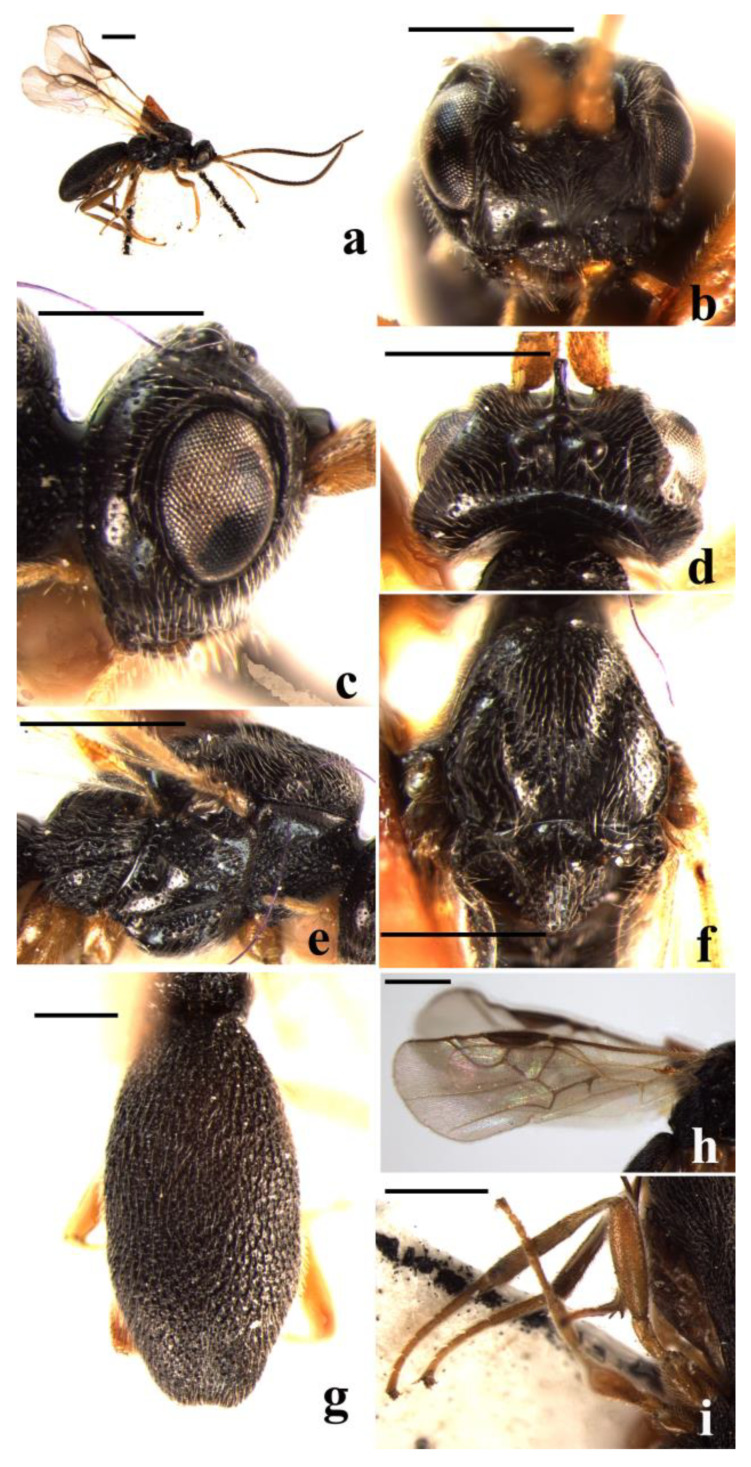
*Schizoprymnus punctiscutellaris* sp. nov., holotype, ♀. (**a**) Habitus, lateral aspect; (**b**) head, frontal aspect; (**c**) head, lateral aspect; (**d**) head, dorsal aspect; (**e**) mesosoma, lateral aspect; (**f**) mesoscutum, dorsal aspect; (**g**) carapace, dorsal aspect; (**h**) fore wing; (**i**) hind leg, lateral aspect. Scale bar: 1 mm.

1. Rim of carapace weakly denticulate posteriorly kong ····················*S. chouwen* (Chou and Hsu, 1996)
- Rim of carapace simple, without small teeth posteriorly····················2
2. Third tergite and precoxal sulcus completely smooth····················*S. calvus* (Chou and Hsu, 1996)
- Third tergite weakly to strongly sculptured, at most partly smooth apically or medially; precoxal sulcus at least weakly sculpture····················3
3. Frons with large lamellate carina medially (Figure 5d)····················4
- Frons without large lamellate carina medially (Figure 4d) ····················8
4. Length of Malar space about 0.7 times basal width of mandible; hind coxa rugose dorso-basally; [clypeus densely punctate; frons punctate laterally; precoxal sulcus densely punctate]····················*S. lienhuachihensis* (Chou and Hsu, 1996)
- Length of Malar space 1.2–2.3 times basal width of mandible; hind coxa striate or superficially rugulose dorso-basally····················5
5. Temple 1.0–1.3 times longer than eye in dorsal view (Figure 3d); frons reticulate-rugose laterally····················6
- Temple 0.7 times as long as eye in dorsal view (Figure 5d); frons laterally tuberculate and punctate; (vertex punctate, with longitudinal medial groove; scutellum convex and densely punctate)····················*S. punctiscutellaris* sp. nov.
6. Clypeus punctate, its ventral margin truncate; vein cu-a of fore wing postfurcal····················7
- Clypeus faintly rugose, convex ventrally, not sharply pointed ventromedially; vein cu-a of fore wing interstitial; (antenna 25–27 segmented; precoxal sulcus rugose-reticulate) ····················*S. borpian* (Chou and Hsu, 1996)
7. Length of Malar space 2.3 times basal width of mandible; temple as long as eye in dorsal view; clypeus punctate····················*S. liui* sp. nov.
- Length of Malar space 1.2 times basal width of mandible; temple 1.2–1.3 times longer than eye in dorsal view; clypeus rugose-punctate····················*S. pallidipennis* (Herrich-Schäffer,1838)
8. Carapace postero-ventrally distinctly incurved····················*S. imitatus* Papp, 1993
- Carapace postero-ventrally not or at most weakly incurved····················9
9. Vein 1-R1 of forewing shorter than or equal to pterostigmal length (Figure 2g)····················10
- Vein 1-R1 of forewing distinctly longer than pterostigmal length (Figure 1g)····················24
10. Clypeus concave apico-ventrally, sharply pointed medio-ventrally····················11
- Clypeus almost straight to convex, not sharply pointed medio-ventrally····················13
11. Metasoma 1.7 times as long as wide; carapace apico-ventrally weakly incurved····················*S. shan* (Chou and Hsu, 1996)
- Metasoma 1.3 times as long as wide; carapace apico-ventrally almost not incurved····················12
12. Metasoma distinctly higher posteriorly than anteriorly in lateral view; antenna 23 segmented; hind coxa rugose basally; vein 1-R1 of forewing almost as long as pterostigmal length····················*S. hui* (Chou and Hsu, 1996)
- Metasoma indistinctly higher posteriorly than anteriorly in lateral view; antenna 19 segmented; hind coxa smooth basally; vein 1-R1 of forewing shorter than pterostigmal length····················*S. chiu* (Chou and Hsu, 1996)
13. Antenna 36 segmented; ovipositor sheath 0.65 times as long as forewing····················*S. bicolor* (Chou and Hsu, 1996)
- Antenna 16–27 segmented; ovipositor sheath 0.2–0.6 times as long as forewing····················14
14. Apical rim of carapace only slightly emarginated medially ····················*S. plenus* (Chou and Hsu, 1996)
- Apical rim of carapace moderately to strongly emarginated or semicircularly emarginated medially····················15
15. Distance between tentorial pits 0.8 times distance from pit to eye; apical rim of carapace semicircularly emarginated····················*S. loi* (Chou and Hsu, 1996)
- Distance between tentorial pits 1.0–2.0 times distance from pit to eye; apical rim of carapace moderately to strongly emarginated····················16
16. Frons laterally, vertex and temple smooth····················17
- Frons laterally and vertex punctate or densely reticulate-punctate; temple punctate····················19
17. Precoxal sulcus shallow and smooth; scutellum smooth····················*S. glabratus* sp. nov.
- Precoxal sulcus deep and sculptured; scutellum sculptured····················18
18. Distance between tentorial pits equal to distance from pit to eye; precoxal sulcus reticulate-punctate; hind coxa dark reddish brown····················*S. telengai* Tobias, 1976
- Distance between tentorial pits 1.6 times distance from pit to eye; precoxal sulcus punctate-crenulate, posteriorly reticulate-punctate; hind coxa yellowish brown····················*S. septentrionalis* sp. nov.
19. Length of body 3.2 mm; clypeus punctate, its ventral margin rounded; vein cu-a of forewing interstitial; ovipositor sheath 0.2 times as long as forewing ····················*S. subspinosus* sp. nov.
- Length of body 1.9–2.4 mm; clypeuspunctate or rugose-reticulate, almost straight ventrally; vein cu-a of forewing postfurcal; ovipositor sheath 0.33–0.5 times as long as forewing····················20
20. Hind coxa smooth; body dark brown····················21
- Hind coxa sculptured; body black····················22
21. Ovipositor sheath 0.33 times as long as forewing; apical rim of carapace moderately emarginated medially····················*S. distinctus* (Chou and Hsu, 1996)
- Ovipositor sheath 0.48 times as long as forewing; apical rim of carapace strongly emarginated medially····················*S. tungpuensis* (Chou and Hsu, 1996)
22. Metasomal carapace 1.7 times longer than wide in dorsal view; length of body 2.4 mm; antenna 16–18 segmented····················*S. ketiao* (Chou and Hsu, 1996)
- Metasomal carapace 1.3–1.4 times longer than wide in dorsal view; length of body 1.9–2.2 mm; antenna 20–23 segmented ····················23
23. Third tergite rugose-reticulate posteriorly; apical rim of carapace strongly emarginated; hind coxa black····················*S. beitun* (Chou and Hsu, 1996)
- Third tergite smooth posteriorly; apical rim of carapace moderately emarginated; hind coxa largely brownish yellow····················*S. robustus* (Chou and Hsu, 1996)
24. Ovipositor sheath distinctly shorter than carapace····················25
- Ovipositor sheath distinctly longer than carapace····················27
25. Metasoma in dorsal view constricted posteriorly; distance between tentorial pits 0.6–0.7 times distance from pit to eye····················*S. fessus* (Chou and Hsu, 1996)
- Metasoma in dorsal view rather rounded posteriorly; distance between tentorial pits 0.75–0.85 times distance from pit to eye····················26
26. Clypeus distinctly rugose ventrally; precoxal sulcus rugose-reticulate····················*S. chunji* (Chou and Hsu, 1996)
- Clypeus sparsely punctate ventrally; precoxal sulcus punctate····················*S. curvatus* (Chou and Hsu, 1996)
27. Clypeus convex ventrally; antenna about 31 segmented····················*S. carinatus* sp. nov.
- Clypeus almost straight ventrally; antenna 17–25 segmented····················28
28. Frons smooth laterally; apical rim of carapace weakly emarginated····················*S. parvidentatus* sp. nov.
- Frons punctate to densely punctate laterally; apical rim of carapace moderately emarginated····················29
29. Ovipositor sheath 0.55 times as long as forewing; carapace 1.2 times longer than wide in dorsal view; antenna 23–25 segmented····················*S. kueichia* (Chou and Hsu, 1996)
- Ovipositor sheath 0.7 times as long as forewing; carapace 1.5 times longer than wide in dorsal view; antenna 17–20 segmented····················S. ovatus (Chou annd Hsu, 1996)

### 3.2. Species Descriptions

#### 3.2.1. *Schizoprymnus carinatus* Yan & Chen, sp. nov. (Figure 1)

**Material examined**. Holotype: ♀, China, Guizhou Prov., Kuankuoshui Natural Reserve, Qinggangtangzhen, 8.VI.2010, Pu Tang, No.201000376 (ZJUH). Paratype: 1♀, China, Guizhou Prov., Kuankuoshui Natural Reserve, Xiangzhangwan, 2.VI.2010, Jiangli Tan, No.201002160 (ZJUH).

**Description**. Female. Body length (excluding ovipositor sheath) 3.5 mm; length of extended part of ovipositor 2.3 mm; fore wing length 3.4 mm.

Head. Antennomeres 31, length of third antennomere almost equal to length of fourth antennomere, length of the third, fourth, and penultimate antennomeres are 3.3, 3.3, and 1.5 times their width, respectively; length of the maxillary palp is 0.6 times the height of the head; POL:OD:OOL = 9:7:15; occipital carina is complete, distinct, and arched medio-dorsally; frons is weakly concave, densely rugose-reticulate, medially with a distinct median carina; in dorsal view length of eye equal to length of temple; vertex densely rugose-reticulate largely, only punctate near occipital carina; temple punctate, rugose-punctate near mandible; face densely reticulate-rugose; clypeus convex and coarsely rugose-reticulate, its ventral margin rounded; malar suture absent; length of the Malar space equal to basal width of mandible; tentorial pits distinct, distance between pits 1.5 times distance from pit to eye. 

Mesosoma. Length of mesosoma is 1.5 times its height; pronope wide, with a medio-longitudinal carina; side of pronotum crenulate submedially, posteriorly punctate, dorsally and medially nearly smooth, ventrally reticulate-punctate; mesosternal suture distinctly crenulate; prepectal carina complete, strong; precoxal sulcus coarsely rugose-reticulate; notauli crenulate, reticulate-rugose posteriorly and with a median carina; mesoscutal lobes densely punctate; scutellar sulcus wide and rugose, with one median carina; scutellum convex, nearly smooth and shiny anteriorly and rugose-punctate laterally and posteriorly; surface of propodeum coarsely reticulate-rugose, basally reticulate-punctate, with a short medio-longitudinal carina and a transverse carina subanteriorly. 

Wings. Fore wing: pterostigma is about 3.0 times as long as wide; 1-R1 1.2 times as long as pterostigma; r:3-SR + SR1:2-SR = 12:75:20; 1-SR + M smoothly curved; SR1 strongly curved; cu-a postfurcal; m-cu antefurcal. Hind wing: 1-M:1r-m = 30:21; M + CU:1-M = 30:41; cu-a straight. 

Legs. Hind coxa smooth. Tarsal claw with a lobe. Length of femur, tibia, and basitarsus of hind leg are 3.2, 6.7, and 5.0 times their width, respectively.

Metasoma. In dorsal view, carapace 1.4 times as long as wide. Surface of the carapace is densely reticulate-rugose, its dorsal carinae only basally distinct. First and second sutures are present but weakly developed. Carapace not incurved posteriorly. Apical rim of carapace emarginated medially. Length of ovipositor sheath is 0.7 times the fore wing, 1.9 times the hind tibia, and 1.4 times the length of the carapace.

Colour. Black. Antenna, clypeus, and mandible are dark reddish brown. Basal one-third of hind tibia whitish yellow; apical two-thirds of hind tibia, hind tarsus, telotarsus of fore and middle tarsi, pterostigma and ovipositor sheath dark brown; remainder of legs, palpi, and tegulae yellowish brown. Prothorax and notauli reddish brown. Wing membrane is faintly infuscated with veins from light brown to dark brown.

**Variation**. Length of the body 3.2–3.5 mm. Length of extended part of ovipositor sheath 1.8–2.3 mm. One female with basal one-third of hind tibia brown and metasoma laterally dark brown.

**Diagnosis**. This new species is similar to *S. oncogena* Belokobylskij, 1994, but differs in the distance between tentorial pits 1.5 times distance from pit to eye (2.5 times in *S. oncogena*); the ventral margin of clypeus rounded (its ventral margin truncate in *S. oncogena*); and the first and second sutures present but weakly developed (first suture distinct; second suture present, but weakly developed medially in *S. oncogena*).

**Distribution**. China (Guizhou).

**Host**. Unknown.

**Etymology**. Name refers to “*carinatus*” (Latin for carina), because its pronope has a medio-longitudinal carina.

#### 3.2.2. *Schizoprymnus glabratus* Yan and Chen, sp. nov. (Figure 2)

**Material examined**. Holotype: ♀, China, Zhejiang Prov., West Tianmu Mountain, Xianrending, 29.VII.1998, Mingshui Zhao, No.993261 (ZJUH). Paratypes: 2♀♀1♂, China, Zhejiang Prov., West Tianmu Mountain, Xianrending, 23.VII.1993, Mingshui Zhao, No.991957, 991919, 991944 (ZJUH); 4♀♀4♂♂, China, Zhejiang Prov., West Tianmu Mountain, Xianrending, 29.VII.1998, Mingshui Zhao, No.993273, 993634, 993638, 993642, 993249, 993306, 993265, 993440 (ZJUH); 1♀, China, Zhejiang Prov., West Tianmu Mountain, Xianrending, 12.VII.1999, Mingshui Zhao, No.20003478 (ZJUH); 1♂, China, Zhejiang Prov., West Tianmu Mountain, Xianrending, 20.VII.1998, Mingshui Zhao, No.992878 (ZJUH); 1♂, China, Zhejiang Prov., West Tianmu Mountain, Xianrending, 9.VIII.1998, Mingshui Zhao, No.994130 (ZJUH); 2♀♀, China, Zhejiang Prov., West Tianmu Mountain, Xianrending, 24.VIII.1998, Mingshui Zhao, No.994749, 994757 (ZJUH); 1♀, China, Zhejiang Prov., West Tianmu Mountain, 16.VIII.1998, Xuexin Chen, No.998121 (ZJUH); 1♀, China, Zhejiang Prov., West Tianmu Mountain, Xianrending, 16.VIII.1999, Xuexin Chen, No.997227 (ZJUH); 1♀, China, Zhejiang Prov., West Tianmu Mountain, Xianrending, 18.VIII.1999, Yun Ma, No.997609 (ZJUH); 4♀♀, China, Zhejiang Prov., West Tianmu Mountain, Xianrending, 18.VIII.1999, Yafen Yang, No.997816, 997820, 997698, 997699 (ZJUH); 2♀♀, China, Zhejiang Prov., West Tianmu Mountain, Xianrending, 18.VIII.1999, Yun Ma, Yafen Yang, No.998029, 998049 (ZJUH); 1♀, China, Zhejiang Prov., Anji, Longwangshan, 24.VI.1998, Qiang Li, No. 963092 (ZJUH); 1♀, China, Zhejiang Prov., Longquan, Fengyangshan, 10.VIII.2003, Jingxian Liu, No.20048218 (ZJUH); 1♀, China, Zhejiang Prov., Linan, Qingliangfeng, 10.VIII.2005, Hongying Zhang, No.200607449 (ZJUH); 1♀, China, Fujian Prov., Huanggangshan, 30.VII.1985, Minghui Liu, No.20004122 (ZJUH).

**Description**. Female. Body length (excluding ovipositor sheath) 2.5 mm; length of extended part of ovipositor sheath 0.9 mm; fore wing length 2.7 mm.

Head. Antennomeres 27, length of the third antennomere is 0.9 times the fourth antennomere; length of the third, fourth, and penultimate antennomeres are 3.8, 4.0, and 1.7 times their width, respectively. Length of the maxillary palp 0.9 times height of head. POL:OD:OOL = 7:5:10. Occipital carina is complete, distinct; frons is weakly concave, smooth, and shiny, medio-ventrally striate with a distinct median carina; in dorsal view, the length of the eye is 1.1 times the length of the temple; vertex and temple are smooth; face is punctate, medially weakly convex; clypeus coarsely punctate, its ventral margin truncate; malar suture present; length of Malar space equal to basal width of mandible. Tentorial pits distinct, distance between pits 2.0 times distance from pit to eye.

Mesosoma. Length of mesosoma is 1.3 times its height. Pronope is deep. Side of pronotum antero-medially and ventro-posteriorly crenulate, antero-ventrally striate, remainder smooth. mesosternal suture distinctly crenulate; prepectal carina complete; precoxal sulcus shallow, smooth and shiny. Notauli narrow and crenulate. Mesoscutal lobes sparsely punctate and shiny. Scutellar sulcus wide and rugose, with one median carina. Scutellum convex, smooth, and shiny. Surface of propodeum reticulate-punctate, anteriorly with a medio-longitudinal carina and a protruding transverse carina.

Wings. Fore wing: pterostigma about 3.0 times longer than wide; 1-R1 as long as pterostigma; r:3-SR + SR1:2-SR = 8:60:18; 1-SR + M smoothly curved; SR1 strongly curved; cu-a postfurcal; m-cu antefurcal. Hind wing: 1-M:1r-m = 20:8; M + CU:1-M = 23:20; cu-a straight.

Legs. Hind coxa striate baso-dorsally. Tarsal claw simple. Length of femur, tibia, and basitarsus of hind leg are 4.3, 7.0, and 6.7 times their width, respectively.

Metasoma. In dorsal view carapace 1.5 times as long as wide. Surface of carapace very densely reticulate-striate, its dorsal carinae distinct extreme basally. First suture visible and second suture present but weakly developed. Carapace not incurved posteriorly. Apical rim of carapace emarginated. Length of ovipositor sheath is 0.3 times the fore wing, 1.2 times the hind tibia, and 0.8 times the length of the carapace.

Color. Black. Basal four antennomeres of antenna (remainder dark brown) and mandible largely yellowish brown. Palpi, tegulae, and legs (except apical half of hind tibia and hind tarsus brown) yellow. Metasomal sternites, pterostigma, and ovipositor sheath brown. Wing membrane faintly brown with veins from light brown to brown.

**Variation**. Antennomeres 27–29. Length of the fore wing is 2.7–3.2 mm, of body 2.5–3.0 mm. In some females, precoxal sulcus with some punctures.

Male. Similar to females in color, sculpture, and morphometrics except sexual characters.

**Diagnosis**. This new species is similar to *S. ambiguus* (Nees, 1816), but differs in the number of antennomeres 27–29 (antenna with 19–22 antennomeres in *S. ambiguus*); the ovipositor sheath is 0.8 times as long as the carapace (0.5 times in *S. ambiguus*); and the carapace is not incurved posteriorly in dorsal view (incurved in *S. ambiguus*).

**Distribution**. China (Zhejiang, Fujian).

**Host**. Unknown.

**Etymology**. Name refers to “*glabratus*” (Latin for smooth) because its scutellum is smooth and shiny.

#### 3.2.3. *Schizoprymnus liui* Yan & Chen, sp. nov. (Figure 3)

**Material examined**. Holotype: ♀, China, Ningxia Prov., Liupanshan, Hongxialinchang (Malaise trap), 2.VII.2000, Jingxian Liu, No.200902287 (ZJUH). Paratypes: ♀, China, Guizhou Prov., Kuankuoshui Natural Reserve, Shilingou, 3–5.VI.2010, Yuhan Qian, No.201003577 (ZJUH); 1♀, China, Guizhou Prov., Kuankuoshui Natural Reserve, Qinggangtangzhen, 8.VI.2010, Pu Tang, No.201000308 (ZJUH); 1♀, China, Guizhou Prov., Kuankuoshui Natural Reserve, Qinggangtangzhen, 8.VI.2010, Jie Zeng, No.201003734 (ZJUH).

**Description**. Female. Body length (excluding ovipositor sheath) 3.2 mm; length of the extended part of the ovipositor sheath 0.8 mm; fore wing length 2.8 mm.

Head. Antennomeres 30, length of the third antennomere is 0.9 times the fourth antennomere; the length of the third, fourth, and penultimate antennomeres are 3.2, 3.4, and 1.0 times their width, respectively; length of the maxillary palp is 0.8 times the height of the head; POL:OD:OOL = 9:5:15; occipital carina is complete, distinct, and arched medio-dorsally; frons is distinctly concave and punctate medially and with a strongly protruding lamella, laterally, tuberculate, reticulate-rugose; in dorsal view, length of eye almost equal to length of temple; vertex sparsely punctate and shiny, with a weakly longitudinal groove; temple sparsely punctate and shiny, reticulate-punctate near mandible; face rugose-reticulate, medially with a medio-longitudinal carina; malar suture is present; clypeus is convex and punctate, its ventral margin truncate; length of the Malar space is 2.3 times the basal width of the mandible; tentorial pits are distinct, and the distance between the pits is 0.5 times the distance from pit to eye.

Mesosoma. Length of mesosoma is 1.7 times its height; pronope is deep; side of pronotum medially and posteriorly coarsely crenulate, dorsally almost smooth, ventrally reticulate; mesosternal suture deep and crenulate; prepectal carina complete, strong; precoxal sulcus rugose-reticulate; notauli wide, crenulate, posteriorly rugose-punctate, and without a median carina; mesoscutal lobes are finely punctate; scutellar sulcus is deep, wide, with one median carina and several lateral carinae; scutellum convex, medially smooth and laterally rugose-punctate; surface of propodeum densely reticulate-rugose, basally punctate, with a longitudinal median carina.

Wings. Fore wing: pterostigma about 3.0 times as long as wide; 1-R1 0.9 times as long as pterostigma; r:3-SR + SR1:2-SR = 7:57:21; 1-SR + M slightly curved; SR1 strongly curved; cu-a postfurcal; m-cu antefurcal. Hind wing: 1-M:1r-m = 22:11; M + CU:1-M = 29:22; cu-a slightly curved.

Legs. Hind coxa is largely smooth, striate dorsally. Tarsal claws are simple. Length of femur, tibia, and basitarsus of the hind leg are 4.6, 11, and 8.0 times their width, respectively.

Metasoma. In dorsal view, the carapace is 2.2 times as long as wide. Surface of carapace is very densely reticulate-rugose, its dorsal carinae distinct only narrowly basally; first and second sutures are absent; the carapace is distinctly incurved posteriorly; the apical rim of the carapace is emarginated; the length of ovipositor sheath is 0.3 times the fore wing, 0.6 times the hind tibia, and 0.4 times the length of the carapace.

Color. Black. Palpi, tegulae, basal five antennomeres of the antenna (remainder dark reddish brown) and legs (except apical one-third of hind tibia, which is dark brown) are yellowish brown. Mandible and clypeus are dark yellowish brown. Pterostigma is brown. Sternites of metasoma and ovipositor sheath are dark reddish brown. Wing membrane is faintly brown. Veins are from light brown to brown.

Male. Unknown.

**Variation**. The paratypes have 25, 26, and 27 antennomeres, the length of the Malar space is 1.7–2.3 times the basal width of themandible, the length of the fore wing is 3.1–3.4 mm, of the body 3.7–4.2 mm; legs (except tarsus and hind tibia) are yellowish brown; tarsus and hind tibia are largely dark brown in one paratype.

**Diagnosis**. This new species is similar to *S. spinosus* Belokobylskij, 1994, but differs in that the frons is punctate medially and with a strongly protruding lamella, laterally strongly convex, tuberculate, reticulate-rugose (frons is punctate-rugose, medially with a long blunt protuberance in *S. spinosus*); the distance between tentorial pits is 0.5 times the distance from pit to eye (0.8 times in *S. spinosus*); and the length of the Malar space is 1.7–2.3 times the basal width of the mandible (1.1 times in *S. spinosus*).

**Distribution**. China (Ningxia, Guizhou).

**Host**. Unknown.

**Etymology**. It is named after the collector of the holotype, Dr. Jing-xian Liu.

#### 3.2.4. *Schizoprymnus parvidentatus* Yan and van Achterberg, sp. nov. (Figure 4)

**Material examined**. Holotype: ♀, China, Guizhou Prov., Kuankuoshui Natural Reserve, 8.VI.2010, Jiangli Tan, No.201002120 (ZJUH).

**Description**. Female. Body length (excluding ovipositor sheath) 2.4 mm; length of extended part of ovipositor sheath 1.2 mm; fore wing length 2.8 mm.

Head. Antennomeres 21, the length of the third antennomere is 0.8 times the length of the fourth antennomere; the length of the third, fourth, and penultimate antennomeres are 2.4, 3.0, and 1.0 times their width, respectively. Length of maxillary palp is 0.6 times the height of the head; POL:OD:OOL = 8:5:10; the occipital carina is complete and distinct; frons is weakly concave, largely smooth and shiny, medio-ventrally striate and with a distinct median carina; in dorsal view, the eye 1.1 times longer than the temple; vertex and temple are smooth and shiny; face is punctate, with some aciculae near the antennal sockets, only slightly elevated centrally; clypeus is coarsely punctate, its ventral margin truncate; malar suture is distinct; the length of the Malar space is equal to the basal width of the mandible; anterior tentorial pits are distinct, and the distance between the pits is 2.5 times the distance from pit to eye.

Mesosoma. Length of the mesosoma is 1.3 times its height. Pronope is wide and deep; the side of the pronotum crenulate medially, postero-dorsally striate, ventrally finely rugose, remainder is smooth; mesosternal suture is distinctly crenulate; prepectal carina is complete, strong; precoxal sulcus is reticulate-punctate; notauli is narrow and crenulate, with a short median carina posteriorly; mesoscutal lobes are sparsely punctate; scutellar sulcus is deep and rugose, with five distinct carinae; scutellum is convex, smooth, and shiny; the surface of the propodeum is reticulate-punctate, basally punctate, with a short medio-longitudinal carina and an irregular transverse carina subanteriorly, lateral tubercle is wide and medium-sized.

Wings. Fore wing: pterostigma about is 3.0 times as long as wide. 1-R1 is 1.3 times as long as the pterostigma; r:3-SR + SR1:2-SR = 10:80:14. 1-SR + M smoothly curved. SR1 is strongly curved. cu-a is distinctly postfurcal, m-cu antefurcal. Hind wing: 1-M:1r-m = 26:13. M + CU:1-M = 26:27. cu-a slightly curved.

Legs. Hind coxa is baso-dorsally striate and remainder is largely smooth. Tarsal claw with a small lobe. Length of femur, tibia, and basitarsus of hind leg are 3.3, 8.2, and 5.0 times their width, respectively.

Metasoma. In dorsal view, the carapace is 1.3 times longer than wide; in lateral view, 2.8 times longer than its maximum height. Surface of the carapace is densely punctate-striate, but the third tergite is smooth and shiny medially, its dorsal carinae only basally distinct. First and second sutures are visible laterally but obscure medially. The carapace is not incurved posteriorly, apical 0.1 of carapace closed and carapace cavity 0.9 times as long as carapace. Apical rim of the carapace is weakly emarginated. Length of ovipositor sheath is 0.5 times the fore wing, 1.5 times the hind tibia, and 1.3 times the length of the carapace.

Color. Black. Mandible is basally dark reddish brown. Middle tibia, fore and middle tarsi are dark yellowish brown; apical two-thirds of the hind tibia and hind tarsus are dark brown; remainder of legs yellowish brown. Tegulae and pterostigma are dark brown. Wing membrane faintly infuscated, and veins are brown or dark brown.

Male. Unknown.

**Diagnosis**. This new species is similar to *S. ungularis* Belokobylskij, 1994 from Far East Russia, but differs by having the third antennomere 2.4 times longer than wide (3.3 times in *S. ungularis*), the length of the Malar space is equal to the basal width of the mandible (0.7 times), the distance between the anterior tentorial pits is 2.5 times longer than distance between the pit and the eye (1.2–1.3 times), the propodeal lateral tubercle is medium-sized and wide (long and narrow), the vein 1-R1 of the fore wing is 1.3 times longer than pterostigma (subequal), the carapace 1.3 times longer than wide in dorsal view (1.6–1.7 times), the middle and hind coxae are yellowish brown (darkened), and the tegulae are blackish (dark reddish brown). It is also similar to *S. querculus* Papp, 1989 from Korea because of the similar size of the Malar space, relative distance between the anterior tentorial pits and the distance to the eye, and comparatively short third antennomere, but differs by having the carapace 1.3 times longer than wide in dorsal view (1.7–1.8 times in *S. querculus*), the four basal antennomeres black (yellow), the length of hind femur 3.3 times longer than wide (2.6–2.7 times), and the vein 1-R1 1.3 times longer than pterostigma (0.7 times).

**Distribution**. China (Guizhou).

**Host**. Unknown.

**Etymology**. The name is derived from “*parvus*” (Latin for “small”) and “*dentatus*” (Latin for “with tooth”) because the tarsal claws have a small lobe.

#### 3.2.5. *Schizoprymnus punctiscutellaris* Yan and Chen, sp. nov. (Figure 5)

**Material examined**. Holotype: ♀, China, Zhejiang Prov., Tianmushan, Xianrending (Malaise trap), 20.VII.1998, Mingshui Zhao, No.992702 (ZJUH). Paratype: 1♀, China, Zhejiang Prov., Tianmushan, Xianrending (Malaise trap), 4.VII.1998, Mingshui Zhao, No.992284 (ZJUH).

**Description**. Female. Body length (excluding ovipositor sheath) is 3.6 mm; length of extended part of ovipositor sheath is 0.5 mm; fore wing length is 3.3 mm.

Head. Antennomeres 31, length of the third antennomere is 0.9 times the fourth antennomere, length of the third, fourth, and penultimate antennomeres are 2.8, 3.3, and 1.3 times their width, respectively; length of maxillary palp is 0.7 times the height of the head; POL:OD:OOL = 9:7:13; occipital carina is complete, distinct, and arched medio-dorsally; frons is distinctly concave, medially nearly smooth, and with a strongly protruding lamella, laterally strongly convex, tuberculate, punctate; in dorsal view, the length of the eye is 1.4 times the temple; vertex is punctate, with longitudinal groove; the temple is sparsely punctate and shiny, rugose-punctate near mandible; face is punctate; clypeus is punctate-rugose, its apical round; length of the Malar space is 1.2 times the basal width of the mandible. Tentorial pits are distinct, and the distance between pits is almost equal to the distance from pit to eye.

Mesosoma. Length of the mesosoma is 1.5 times its height; pronope is triangular; side of pronotum is medially coarsely punctate-crenulate, dorsally punctulate, posteriorly and ventrally reticulate-punctate; mesosternal suture is deep and crenulate; prepectal carina is complete, strong; precoxal sulcus is densely punctate; notauli is complete, crenulate, with a median carina posteriorly; mesoscutal lobes are finely and densely punctate; scutellar sulcus is deep, wide, with one median carina and several lateral carinae; scutellum is convex and densely punctate; surface of propodeum is reticulate-rugose, but anteriorly punctate, with a medio-longitudinal carina and an irregularly transverse carina.

Wings. Fore wing: pterostigma is 3.0 times as long as wide; 1-R1 0.8 times as long as pterostigma; r:3-SR + SR1:2-SR = 10:47:17; 1-SR + M curved; SR1 strongly curved; cu-a postfurcal; m-cu distinctly antefurcal. Hind wing: 1-M:1r-m = 18:10; M + CU:1-M = 31:18; cu-a weakly curved.

Legs. Hind coxa are largely smooth, striate dorsally. Tarsal claws are simple. Length of femur, tibia, and basitarsus of the hind leg are 4.0, 8.3, and 6.2 times their width, respectively.

Metasoma. In dorsal view, the carapace is 2.0 times as long as wide. Surface of the carapace is very coarsely and densely reticulate-rugose, its dorsal carinae distinct only basally; first and second sutures are absent; apical rim of the carapace is emarginated; the length of the ovipositor sheath is 0.1 times the fore wing, 0.4 times the hind tibia, and 0.3 times the length of the carapace.

Color. Black. Palpi, basal five antennomeres of antenna (remainder dark brown), fore and middle legs are yellow brown. Mandible basally, tegulae, hind leg, and sternites of metasoma are dark yellowish brown. Pterostigma and ovipositor sheath are dark brown. Wing membrane is faintly brown. Veins are from light brown to dark brown.

Male. Unknown.

**Variation.** Antennomeres 29–31. Body length (excluding ovipositor sheath) 2.7–3.6 mm, fore wing length 2.6–3.3 mm.

**Diagnosis.** This new species is similar to *S. spinosus* Belokobylskij, 1994, but differs by having the frons medially nearly smooth and with a strongly protruding lamella, laterally tuberculate and punctate (frons punctate-rugose, medially with a long blunt protuberance in *S. spinosus*); the length of the eye is 1.4 times the temple in dorsal view (0.8 times in *S. spinosus*); and the length of ovipositor sheath is 0.1 times the fore wing (0.3 times in *S. spinosus*). The new species is closely related to *S. liui*; for the differences, see the couplet 7 in the key above.

**Distribution.** China (Zhejiang).

**Host.** Unknown.

**Etymology.** The name is derived from “*punctus*” (Latin for “punctate”) and “*scutellaris*” (Latin for “scutellum”) because its scutellum is densely punctate.

#### 3.2.6. *Schizoprymnus septentrionalis* Yan and Chen, sp. nov. (Figure 6)

**Material examined.** Holotype: ♀, China, Liaoning Prov., Kezuo, Wafangdianshuiku, 7.VIII.2002, Yiping Wang, No.200607019 (ZJUH). Paratypes: 5♀♀26♂♂, China, Liaoning Prov., Kezuo, Wafangdianshuiku, 7.VIII.2002, Yiping Wang, No. 200607006, 200607004, 200607003, 200607023, 200607020, 200607017, 200607010, 200606994, 200607014, 200607016, 200607015, 200607008, 200607024, 200607009, 200607018, 200606999, 200607025, 200607026, 200607000, 200607022, 200607021, 200607005, 200607007, 200607011, 200607012, 200607013, 200606996, 200607002, 200606995, 200607001, 200607027 (ZJUH); 1♂, China, Liaoning Prov., Kezuo, Caochangxiang, 6.VIII.2002, Yiping Wang, No.20030764 (ZJUH); 1♂, China, Liaoning Prov., Linyuan, 8.VIII.2002, Yiping Wang, No.20030652 (ZJUH); 7♀♀1♂, China, Hebei Prov., Xiaowutaishan, Yangjiaping, 20.VIII.2005, Min Shi, No.200607847, No.200607812, 200607821, 2006078247, 200607845, 200607850, 200607849, 200607813 (ZJUH); 9♀♀6♂♂, China, Hebei Prov., Xiaowutaishan, Yangjiaping, 20.VIII.2005, Hongying Zhang, No.200611792, 200611754, 200611741, 200611791, 200611730, 200611753, 200611735, 200611809, 200611772, 200611737, 200611724, 200611811, 200611743 200611780, 200611866 (ZJUH).

**Description.** Female. Body length (excluding ovipositor sheath) is 2.0 mm; length of extended part of ovipositor sheath is 1.2 mm; fore wing length is 2.2 mm.

Head. Antennomeres 19, length of third antennomere is almost equal to the length of the fourth antennomere; length of the third, fourth, and penultimate antennomeres are 3.4, 3.4, and 1.4 times their width, respectively; length of maxillary palp is 0.6 times the height of the head; POL:OD:OOL = 8:5:10; occipital carina is complete, distinct; frons is weakly concave, smooth, and shiny, medially with a distinct median carina; in dorsal view, the length of the eye is 1.1 times the temple; vertex is smooth and shiny, with a weakly longitudinal groove; temple is smooth and shiny; face with small tubercle dorso-medially, punctate medially, with a few aciculae near antennal sockets, only slightly elevated centrally; clypeus punctate, concave apically, sharply pointed medially; malar suture is distinct; length of the Malar space is 1.2 times the basal width of the mandible. Tentorial pits are distinct, and the distance between the pits is 1.6 times the distance from pit to eye.

**Figure 6:**
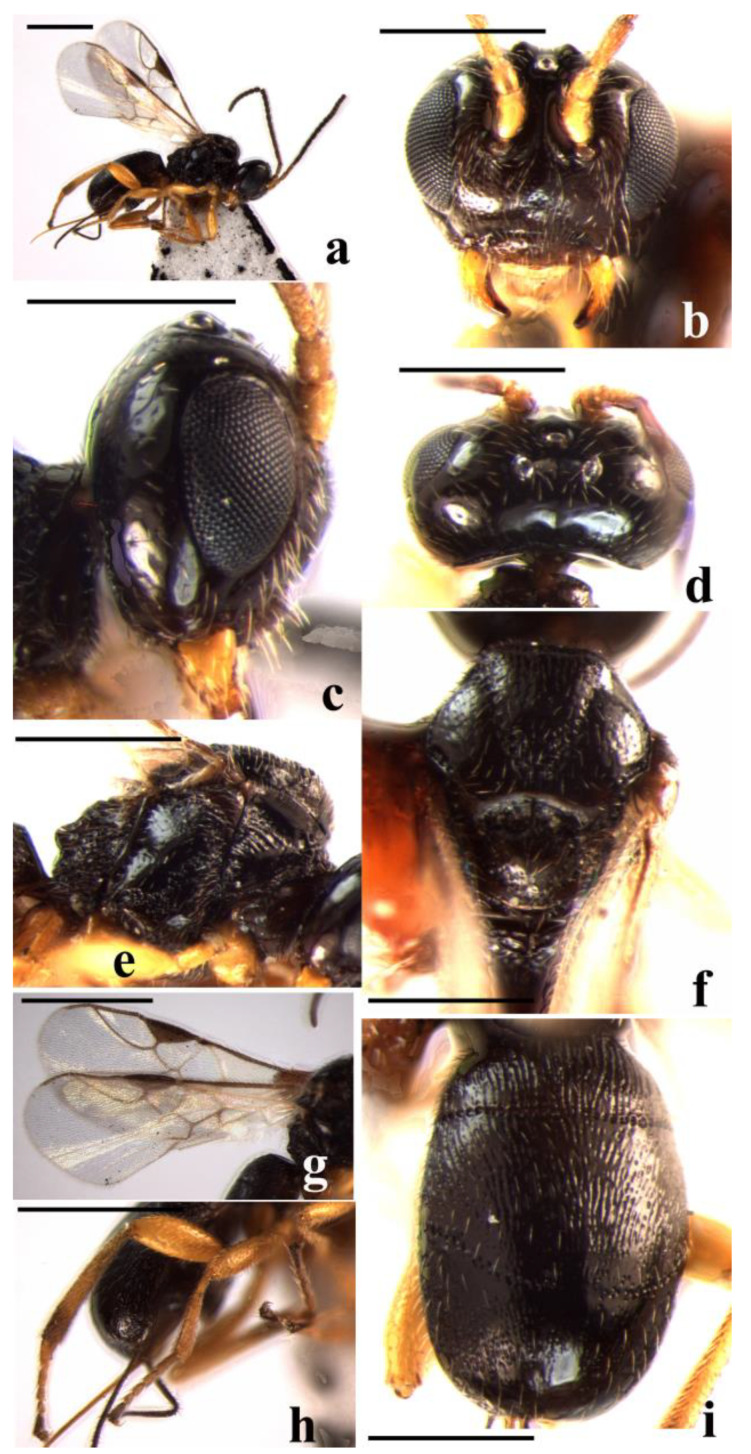
*Schizoprymnus septentrionalis* sp. nov., holotype, ♀. (**a**) Habitus, lateral aspect; (**b**) head, frontal aspect; (**c**) head, lateral aspect; (**d**) head, dorsal aspect; (**e**) mesosoma, lateral aspect; (**f**) mesoscutum, dorsal aspect; (**g**) fore wing; (**h**) hind leg, lateral aspect; (**i**) carapace, dorsal aspect. Scale bar: 1 mm.

Mesosoma. Length of mesosoma is 1.3 times its height; pronope is deep, elliptical-shaped; side of pronotum is posteriorly coarsely crenulate, ventrally punctate-striate, remainder nearly smooth; mesosternal suture distinctly crenulate; prepectal carina is complete, strong; precoxal sulcus is punctate-crenulate, posteriorly reticulate-punctate; notauli narrow and crenulate, posteriorly reticulate-punctate; mesoscutal lobes are finely punctate; scutellar sulcus is deep, punctate, with one strongly median carina; scutellum is convex and sparsely punctate; surface of propodeum is reticulate-rugose.

Wings. Fore wing: pterostigma is about 3.0 times longer than wide; 1-R1 0.8 times as long as pterostigma; r:3-SR + SR1:2-SR = 5:40:10; 1-SR + M smoothly curved; SR1 strongly curved; cu-a postfurcal; m-cu antefurcal. Hind wing: 1-M:1r-m = 22:8; M + CU:1-M = 22:22; cu-a smoothly curved.

Legs. Hind coxa are largely smooth, with some striae dorsally; hind femur is punctate. Tarsal claw with a small lobe. Length of femur, tibia, and basitarsus of hind leg are 2.9, 7.3, and 2.5 times their width, respectively.

Metasoma. In dorsal view, the carapace is 1.3 times as long as wide. First and second tergites are punctate-striate, its dorsal carinae is only basally distinct; the third tergite is punctate medially, laterally punctate-striate; first suture distinct; second suture present, but weakly developed; carapace not incurved posteriorly; apical rim of carapace emarginated; length of ovipositor sheath is 0.6 times the fore wing, 1.8 times the hind tibia, and 1.3 times the length of the carapace.

Color. Black. Palpi is dark brown. Mandible, basal three antennomeres of antenna, and legs (except apical one-third of the hind tibia and the hind tarsus, which are dark brown) are yellowish brown. Tegulae, pterostigma, and ovipositor sheath are dark reddish brown. Wing membrane is faintly infuscate, with veins from light brown or brown.

**Variation.** Antennomeres 19–20. Length of the fore wing is 1.8–2.2 mm, of body 1.8–2.1 mm. In some females, palpi is yellow and the whole metasoma is dark reddish brown. The third tergite is largely smooth with some shallow pits in one female.

Male. Similar to female in color, sculpture, and morphometrics, but body length is 1.7–2.1 mm and in some males third tergite is largely punctate-striate and only medio-posteriorly smooth. Palpi yellow.

**Diagnosis.** This new species is similar to *S. ungularis* Belokobylskij, 1994, but differs in the length of the Malar space 1.2 times the basal width of mandible (0.7 times in *S. ungularis*); the length of ovipositor sheath is 0.6 times the fore wing (0.3 times in *S. ungularis*), and the vein cu-a of the hind wing is smoothly curved (straight in *S. ungularis*).

**Distribution.** China (Liaoning, Hebei).

**Host.** Unknown.

**Etymology.** Name refers to “*septentrionale*” (Latin for northern) because all specimens examined originate from northern China.

#### 3.2.7. *Schizoprymnus subspinosus* Yan and Chen, sp. nov. (Figure 7)

**Material examined.** Holotype: ♀, China, Guizhou Prov., Leigongshan, Balahe, 30.V.2005, Jinxian Liu, No.200605313 (ZJUH). Paratypes: 2♀♀, China, Guizhou Prov., Leigongshan, Balahe, 30.V.2005, Jingxian Liu, No.200605339, 200605318 (ZJUH); 1♀♂, China, Guizhou Prov., Leigongshan, Balahe, 4.V.2005, Hongying Zhang, No.200606142, 200606293 (ZJUH); 1♀, China, Guizhou Prov., Leigongshan, Balahe, 4–5.VI.2005, Jingxian Liu, No.200606531 (ZJUH); 1♀, China, Hunan Prov., Shimen, Hupingdongshanfeng, 14.VII.2009, Pu Tang, No.200901817 (ZJUH).

**Description.** Female. Body length (excluding ovipositor sheath) is 3.2 mm; length of extended part of the ovipositor sheath 0.6 mm; fore wing length is 2.7 mm.

Head. Antennomeres 25, length of third antennomere is almost equal to the length of the fourth antennomere; the length of the third, fourth, and penultimate antennomeres are 3.4, 3.4, and 1.3 times their width, respectively; length of maxillary palp is 0.7 times height of the head; POL:OD:OOL = 9:5:15; occipital carina is complete, distinct; frons is weakly concave, densely reticulate-punctate, medially with a distinct median carina; in dorsal view, the length of the eye is equal to the length of the temple; vertex is reticulate-punctate near stemmaticum, posteriorly nearly smooth; temple is punctate, rugose-punctate near mandible; face is reticulate-punctate, medially with an irregularly longitudinal median carina; clypeus punctate and convex, its ventral margin rounded; Malar suture is present but indistinct; length of the Malar space 1.4 times the basal width of the mandible. Tentorial pits are distinct, and the distance between the pits almost equal to the distance from pit to eye.

**Figure 7:**
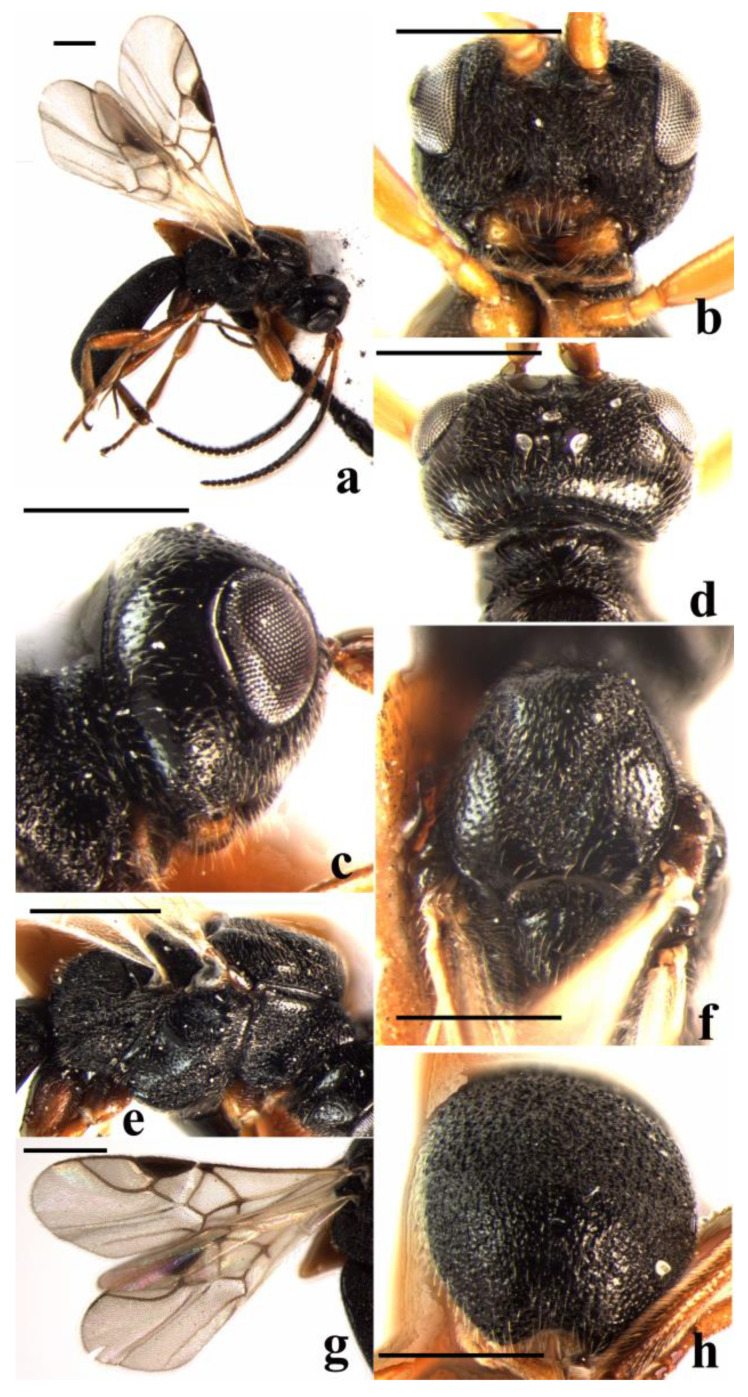
*Schizoprymnus subspinosus* sp. nov., holotype, ♀. (**a**) Habitus, lateral aspect; (**b**) head, frontal aspect; (**c**) head, lateral aspect; (**d**) head, dorsal aspect; (**e**) mesosoma, lateral aspect; (**f**) mesoscutum, dorsal aspect; (**g**) fore wing; (**h**) carapace, posterior aspect. Scale bar: 1 mm.

Mesosoma. Length of the mesosoma is 1.4 times its height; pronope is deep, fan-shaped; side of pronotum is coarsely striate, only dorsally punctate; mesosternal suture is distinctly crenulate; prepectal carina is complete, strong; precoxal sulcus is rugose-reticulate; notauli crenulate, reticulate-rugose posteriorly; middle lobe of mesoscutum is finely punctate medially, laterally reticulate; scutellar sulcus is deep, with one median carina and several lateral carinae; scutellum convex, punctate anteriorly and rugose-punctate posteriorly; surface of the propodeum is reticulate-rugose.

Wings. Fore wing: pterostigma about 3.0 times as long as wide; 1-R1 as long as pterostigma; r:3-SR + SR1:2-SR = 7:55:20; 1-SR + M smoothly curved; SR1 strongly curved; cu-a interstitial; m-cu antefurcal. Hind wing: 1-M:1r-m = 23:10; M + CU:1-M = 38:23; cu-a smoothly curved.

Legs. Hind coxa striate dorsally. Tarsal claw is simple. Length of the femur, tibia, and basitarsus of the hind leg are 3.8, 8.0, and 7.5 times their width, respectively.

Metasoma. In dorsal view, the carapace is 2.0 times as long as wide. Surface of the carapace is very densely reticulate-rugose, and its dorsal carinae is distinct only basally. First and second sutures are present but weakly developed; carapace is weakly incurved posteriorly; posterior rim of the carapace is emarginated; length of ovipositor sheath is 0.2 times the fore wing, 0.6 times the hind tibia, and 0.4 times the length of the carapace.

Color. Black. Clypeus is reddish brown. Palpi is yellowish brown. Mandible largely and basal four antennomeres of antenna are yellow. Coxae dorsally reddish yellow. Fore and middle telotarsi, apical one-third of hind tibia, and hind tarsus are yellowish brown, and the remainder of the legs are yellow. Tegulae, pterostigma, and ovipositor sheath are dark brown. Wing membrane is faintly infuscated and with veins varying from light brown to brown.

**Variation.** Antennomeres 23–25. Length of the body is 3.2–3.7 mm. In some females, the basal four antennomeres of the antenna are yellowish brown.

Male. Similar to female in color, sculpture, and morphometrics except the following: middle lobe of mesoscutum is finely punctate anteriorly, laterally, and posteriorly reticulate and clypeus is dark brown.

**Diagnosis.** This new species is similar to *S. spinosus* Belokobylskij, 1994, but differs by having the frons weakly concave, densely reticulate-punctate, medially with a distinct median carina (frons depressed, punctate-rugose, medially with a long blunt protuberance in *S. spinosus*); the length of the eye is equal to the length of the temple in dorsal view (0.8 times in *S. spinosus*); and the cu-a of the hind wing is smoothly curved (straight in *S. spinosus*).

**Distribution.** China (Guizhou, Hunan)

**Host.** Unknown.

**Etymology.** Name indicates that it is similar to *S. spinosus* Belokobylskij.

#### 3.2.8. *Schizoprymnus telengai* Tobias, 1976 (Figure 8)

*Schizoprymnus telengai*: Belokobylskij, 1998: 481–482.

**Figure 8:**
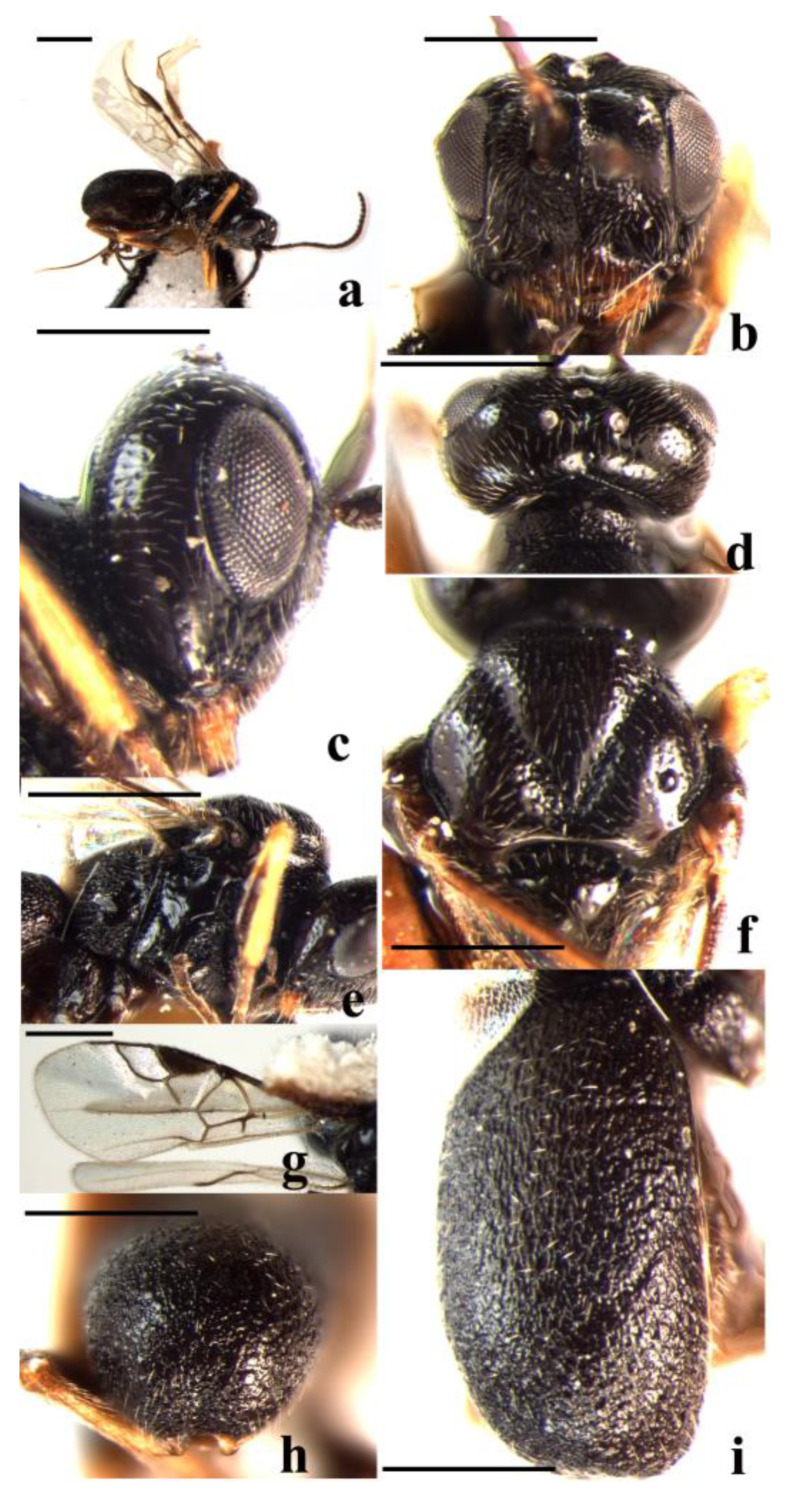
*Schizoprymnus telengai* Tobias, 1976. (**a**) Habitus, lateral aspect; (**b**) head, frontal aspect; (**c**) head, lateral aspect; (**d**) head, dorsal aspect; (**e**) mesosoma, lateral aspect; (**f**) mesoscutum, dorsal aspect; (**g**) fore wing; (**h**) third metasomal tergite apically, dorsal aspect; (**i**) carapace, dorso-lateral aspect. Scale bar: 1 mm.

**Material examined.** 2♀♀1♂, China, Inner Mongolia Prov., 17.VIII.2000, Xuexin Chen, No.200701783, 200701784, 200701781. 1♀1♂, China, Inner Mongolia Prov., 6.VII.2002, Yuanchao Guo, No.20030095, 20030094. 1♀, China, Inner Mongolia Prov., 13.VII.2002, Yuanchao Guo, No.20030193.

**Diagnosis.** Precoxal sulcus is reticulate-punctate; scutellum is sculptured; the temple is as long as the transverse diameter of the eye in dorsal view; vein 1-R1 of fore wing is shorter than pterostigma. Hind coxa are dark reddish brown.

**Distribution.** China (Inner Mongolia), new record; Czech Republic, Slovakia, Kazakhstan, Russia, Turkey, Iran. [7,14]

**Host.** *Titanomalia komaroffi* (Faust) (Coleoptera: Curculionidae) [7]

## 4. Discussion

According to the data from the published references, *Schizoprymnus* was mostly distributed in Taiwan of China, and only one species was recorded in Sichuan of China [7,22]. The discovery of seven new species and one new record in the mainland of China indicates that the species richness of the genus remains underestimated. There is no doubt that further collection and investigation of *Schizoprymnus* taxa are required to understand their real diversity.

## Data Availability

All data are available in this paper.

## References

[1] Papp J. (1984). First survey of the Triaspidini species of the Indo-Australian region (Hymenoptera, Braconidae: Calyptinae) I. The genus *Triaspis* Haliday. Acta Zool. Hung..

[2] Papp J. (1991). First survey of the Triaspidini species of the Indo-Australian region (Hymenoptera, Braconidae: Calyptinae) II. The genus *Schizoprymnus* Foerster, 1. Acta Zool. Hung..

[3] Papp J. (1993). First survey of the Triaspidini species of the Indo-Australian region (Hymenoptera, Braconidae: Calyptinae) 3. The genus *Schizoprymnus* Foerster, 2. Acta Zool. Hung..

[4] Belokobylskij S.A. (1994). To the knowledge of the braconid fauna of the Russian Far East (Hymenoptera, Braconidae): New species of the subfamily Brachistinae. Russ. Entomol. J..

[5] Belokobylskij S.A., Lehr P.A. (1998). Subfam. Brachistinae (Calyptinae), In Opredelitel’ na seko mykh Dal’nego Vostoka Rossii. Tom 4. Setchato Krylo Obraznye, Skorpionnitzy, Pereponchatokrylye. Chast’ 3.

[6] Belokobylskij S.A., Maetô K. (2007). New subgenus of the genus *Schizoprymnus* (Hymenoptera: Braconidae) from Japan, having a unique abdominal carapace structure. Entomol. Sci..

[7] Yu D.S., van Achterberg C., Horstmann K. (2016). Taxapad 2016. World Ichneumonoidea 2016. Taxonomy, Biology, Morphology and Distribution.

[8] Shamim M. (2012). A key to the Indian species of *Schizoprymnus* Foerster (Hymenoptera: Braconidae: Brachistinae) with description of a new Species. Trends Biosci..

[9] Belokobylskij S.A., Taeger A., Dathe H.H., Taeger A., Blank S.M. (2001). Braconidae. Verzeichnis der Hautflügler Deutschlands (Entomofauna Germanica 4).

[10] Belokobylskij S.A., Güçlü C., Özbek H. (2004). A new species of the genus *Schizoprymnus* Förster from Turkey (Hymenoptera: Braconidae, Brachistinae). Zoosystematica Ross..

[11] Chou L.Y., Hsu T.C. (1996). The Braconidae (Hymenoptera) of Taiwan Ⅶ. Subtribe Triaspina. J. Agric. Res. China.

[12] Papp J. (2002). The Braconid wasps (Hymenoptera: Braconidae) of the Fertö-Hanság National Park (NW Hungary). The Fauna of the Fertö-Hanság National Park.

[13] Papp J. (2004). Type specimens of the braconid species by Gy. Szépligeti deposited in the Hungarian Natural History Museum (Hymenoptera: Braconidae). Ann. Hist. Nat. Musei Natl. Hung..

[14] Talebi A.A., Farahani S., Pirouzeh F.Z. (2018). Six new record species of the genus *Schizoprymnus* Förster, 1862 (Hymenoptera: Braconidae, Brachistinae) from Iran. Entomofauna.

[15] Tobias V.I., Medvedev G.S. (1986). Helconinae, Brachistinae. Opredelitel Nasekomych Evrospeiskoi Tsasti SSSR 3, Peredpontdatokrylye 4. Opr. Faune SSSR.

[16] Van Achterberg C. (1993). Illustrated key to the subfamilies of the Braconidae (Hymenoptera: Ichneumonoidea). Zool. Verh..

[17] Van Achterberg C. (1988). Revision of the subfamily Blacinae Foerster (Hymenoptera, Braconidae). Zool. Verh..

[18] Yu Y., Choi S., Kim H. (2019). A new record of parasitoid wasp *Schizoprymnus terebralis* (Hymenoptera: Braconidae) from Korea. Korean J. Appl. Entomol..

[19] Beyarslan A., Deveci R. (2019). Taxonomic studies on the Brachistini (Hymenoptera, Braconidae, Brachistinae) fauna of the Turkish central part of eastern Anatolia region (Bingöl, Bitlis, Muş and Van). Bitlis Eren Univ. J. Sci. Technol..

[20] Chen X.X., van Achterberg C. (2019). Systematics, phylogeny and evolution of braconid wasps: 30 years of progress. Annu. Rev. Entomol..

[21] Shenefelt R.D. (1970). Braconidae 2. Helconinae, Calyptinae, Mimagathidinae, Triaspinae. Hymenopterorum Cat..

[22] Fahringer J. (1935). Schwedisch-chinesische wissenschaftliche Expedition nach den nordwestlichen Provinzen Chinas, 26. Hymenoptera. 4. Braconidae Kirby. Ark. Zool..

[23] Abdinbekova A., Huseynova E., Kerimova I. (2013). Braconidae (Hymenoptera) in the collection of the Institute of Zoology, NAS of Azerbaijan Republic Part III. Subfamilies Helconinae, Brachistinae, Euphorinae, Macrocentrinae (Hymenoptera). Beiträge Entomol. Contrib. Entomol..

[24] Fernandez L., Fernandez E., Moreno M. (2004). Parasitism of *Schizoprymnus* longiseta (Hymenoptera: Braconidae) on Curculio elephas (Coleoptera: Curculionidae) in holm oaks woods in the Montes de Toledo mountains. Ciudad Bolan.

[25] Güçlü C., Özbek H. (2011). A Contribution to the knowledge of the subfamily Brachistinae (Hymenoptera: Braconidae) in Turkey. J. Entomol. Res. Soc..

[26] Van Achterberg C., Hosaka T., Ng Y.F., Ghani I.B. (2009). The braconid parasitoids (Hymenoptera: Braconidae) associated with seeds of Dipterocarpaceae in Malaysia. J. Nat. Hist..

[27] Belshaw R., Quicke D.L.J. (2002). Robustness of ancestral state estimates: Evolution of life history strategy in ichneumonoid parasitoids. Syst. Biol..

[28] Capek M., Hofmann C. (1997). The Braconidae (Hymenoptera) in the collections of the Musée cantonal de zoologie, Lausanne. Litt. Zool..

[29] Ghahari H., Gadallah N.S. (2019). New records of Braconid wasps (Hymenoptera: Ichneumonoidea: Braconidae) from Iran. Entomol. News.

[30] Martin J.C. (1956). A Taxonomic Revision of the Triaspidine Braconid Wasps of Nearctic America (Hymenoptera).

[31] Samin N., Coronado-Blanco J.M., Hosseini A., Fischer M., Sakenin Chelav H. (2019). A faunistic study on the braconid wasps (Hymenoptera: Braconidae) of Iran. Nat. Som..

[32] Szépligeti G. (1904). Hymenoptera. Fam. Braconidae. Genera Insectorum.

